# Effect of a direct sulfonation reaction on the functional properties of thermally-crosslinked electrospun polybenzoxazine (PBz) nanofibers

**DOI:** 10.1039/d0ra01285h

**Published:** 2020-04-07

**Authors:** Ronaldo P. Parreño, Ying-Ling Liu, Arnel B. Beltran, Maricar B. Carandang

**Affiliations:** Department of Chemical Engineering, De La Salle University 2401 Taft Avenue Manila 1004 Philippines arnel.beltran@dlsu.edu.ph; Chemicals and Energy Division, Industrial Technology Development Institute (ITDI), Department of Science and Technology (DOST) Taguig 1631 Philippines ronaldo_parrenojr@dlsu.edu.ph rpparrenojr@yahoo.com; Department of Chemical Engineering, National Tsing Hua University Hsinchu 30013 Taiwan liuyl@mx.nthu.edu.tw; Center for Engineering and Sustainable Development Research, De La Salle University 2401 Taft Avenue Manila 1004 Philippines

## Abstract

Electrospun nanofibers of polybenzoxazines (PBzs) were fabricated using an electrospinning process and crosslinked by a sequential thermal treatment. Functionalization by the direct sulfonation process followed after the post-electrospinning modification treatment. The first stage of experiment determined the effects of varying the concentration of sulfuric acid as the sulfonating agent in the sulfonation reaction under ordinary conditions. The second stage examined the mechanism and kinetics of the sulfonation reaction using only concentrated H_2_SO_4_ at different reaction time periods of 3 h, 6 h, and 24 h. The mechanism of the sulfonation reaction with PBz nanofibers was proposed with only one sulfonic acid (–SO_3_H) group attached to each of the repeating units since only first type substitution in the aromatic structure occurs under this condition. The kinetics of the reaction exhibited a logarithmic correlation where the rate of change in the ion exchange capacity (IEC) with the reaction time increased rapidly and then reached a plateau at the reaction time between 18 h and 24 h. Effective sulfonation was confirmed by electron spectroscopy with a characteristic peak associated with the C–S bond owing to the sulfonate group introduced onto the surface of the nanofibers. ATR-FTIR spectroscopy also confirmed these results for varying reaction times. The SEM images showed that sulfonation has no drastic effects on the morphology and microstructure of the nanofibers but a rougher surface was evident due to the wetted fibers with sulfonate groups attached to the surface. EDX spectra exhibited sulfur peaks where the concentration of sulfonate groups present in the nanofibers is directly proportional to the reaction time. From surface wettability studies, it was found that the nanofibers retained the hydrophobicity after sulfonation but the inherent surface property of PBz nanofibers was observed by changing the pH level of water to basic, which switches its surface properties to hydrophilic. The thermal stability of the sulfonated nanofibers showed almost the same behavior compared to non-sulfonated nanofibers except for the 24 h sulfonation case, which has slightly lower onset temperature of degradation.

## Abstract 2

Functionalization of PBz by electrospinning of nanofibers then thermal crosslinking followed by direct sulfonation process.

## Graphical abstract

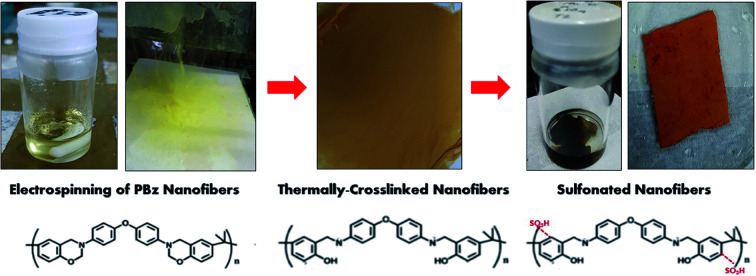

## 1. Introduction

Polybenzoxazines (PBz) are one of the recently developed polymers, which have been attracting research interests in the field of polymers as superior alternatives to thermosetting polymers for high performance applications.^1^ They are a new type of addition-cure phenolic resin, which have the capability to attain good thermal properties and flame retardancy, and exhibit high mechanical performance and molecular design flexibility.^2^ Other remarkable properties of such polymers include chemical inertness, high thermal and temperature stability, flexural strength, low dielectric constant, near zero volumetric change upon curing, and low thermal expansion coefficients.^3–6^ Despite these distinct valuable properties, PBz still has limited applications due to some disadvantages.^3^

PBz, as a polymerization product, plays a special role among the class of highly-crosslinked polymers having benzoxazine (1,3-oxazine cycle condensed with a benzene ring) in their structures.^2,7^ The synthesis of PBz occurs *via* thermally induced ring-opening polymerization (ROP) of the 1,3-benzoxazine monomer, which also results in crosslinked networks.^5,8^ This simple synthesis method for benzoxazine monomers led to the exploitation of addition of new functional groups, which allowed other possible molecular design for PBz.^1^ The addition of functional groups to modify the functionalities of PBz is still being explored as it provides further performance enhancement of the polymer properties. For the polymerization of PBz, functional groups such as thiol compounds were incorporated for its direct modification to reduce the temperature requirement of ROP and provide enhancement of the thermal and mechanical properties.^1^ Another effective approach studied for functionalization was the synthesis of PBz from benzoxazine monomers containing other polymerizable groups^9^ such as ethynyl or phenyl ethynyl,^10^ nitrile,^11^ and propargyl groups.^12^ But, aside from the modification of benzoxazine monomers during the synthetic phase, different strategies of functionalization such as preparation of polymer blends and composites, hybridization with inorganic materials, and chemical incorporation of the benzoxazine structure into the polymer have also been reported.^2^ Several works involve the blending of PBz with other polymers such as rubber,^13^ polycarbonate (PC),^14^ polyurethane (PU),^15^ poly(ε-caprolactone) (PCL),^14^ and poly(*S*-r-diisopropenylbenzene) (SDIB),^16^ which resulted in the modification of the functional properties derived from the synergy of polymer components. In the previous study of Li and Liu,^8^ a composite of polybenzoxazine (PBz)-modified polybenzimidazole (PBI) nanofibers showed enhancement in the mechanical strength using PBz as the crosslinking agent. However, the addition of functional groups during PBz synthesis or the formation of copolymers and composite as the modification methods produced mostly beneficial effects but may also have some negative influences on other properties of the final materials.^3^

Electrospinning process was used for the fabrication of PBz nanofibers. Although electrospinning technique is relatively new for fabricating nanomaterials, it is known to enhance the functionality and versatility of nanofibers, which are brought about by the surface morphologies. The thermal treatment of the nanofibers after electrospinning is a physical method of functionalization, which is an effective post-modification strategy for nanofibers.^17^ In some polymers such as PBz, application of thermal treatment also causes crosslinking reaction in the polymer structure. Chemical treatment along with physical treatment is applied in order to modify or control the surface properties of the electrospun fibers.^18^ For surface modification methods, one of the widely used strategies is to introduce chemically active groups on the surface of the nanofiber-based mat through specific chemical reactions.^17^ Incorporating sulfur-containing functional groups by chemical modification methods have beneficial effects on the physical and chemical properties of the polymer.^19^ Sulfonation is one of the frequently used methods for modification of the properties for better wettability, higher water flux, higher antifouling capacity, better selectivity, and increased solubility in solvents for processing.^20^ The simplest sulfonation process is by direct sulfonation using concentrated sulfuric acid (H_2_SO_4_) as the sulfonating agent conducted under ordinary conditions.^20^

In this study, a different approach of functionalization was explored by the direct sulfonation reaction of electrospun nanofibers that had undergone thermal treatment. Previous studies have investigated the functionalization of PBz by incorporating functional groups during synthesis or by blending and compositing with other polymers and materials. This time, prior to functionalization, PBz was formed into the nanofibers and subjected to successive modification treatment. The PBz nanofibers undergo post-electrospinning thermal treatment, followed by chemical treatment by sulfonation. Thus, this work explores the application of direct sulfonation process to electrospun PBz nanofibers, which, to the best of our knowledge, is the first time such an approach has been used for synthesizing thermally-crosslinked PBz nanofibers. This study examines the nature of chemical interaction between the acid group and the thermally-crosslinked nanofibers, and the effects on the resulting properties, which have not been studied yet. The possible interactions that took place, revealed on studying the mechanism and kinetics of sulfonation, provided a new perspective on the functionalization of PBz nanofibers for applications in separation processes such as in oil–water mixtures.

## 2. Experimental section

### 2.1 Materials

Polybenzoxazine (PBz) was prepared in the lab, as reported in the work by Lin *et al.*^21^ The synthesized PBz had a number-averaged molecular weight of 4040 g mol^−1^.^22^ Concentrated H_2_SO_4_ (analytical grade, 96–97%) was purchased from Aencore and used as received. Dimethylsulfoxide (DMSO) (ACS grade, Echo, 99.9%) and tetrahydrofuran (THF) (inhibitor free high purity, Tedia, 99.8%) were also used as received.

### 2.2 Preparation of electrospun PBz nanofibers

A homogeneous solution of 10 wt% PBz in a mixture of DMSO and THF (1/3) (v/v) was prepared prior to electrospinning. The nanofiber mat was produced using a vertically-aligned electrospinning apparatus consisting of a 10 mL syringe with a needle (ID = 0.8 mm) connected to a syringe pump, a ground electrode, and a high voltage supply (Falco Enterprise Co., Taipei, Taiwan). The needle was connected to the high voltage supply, which generates positive DC voltages up to 40 kV. The electrospinning solution of PBz was placed in the 10 mL syringe, which was ejected through the needle spinneret by a syringe pump with a mass flow rate of 1.00 mL h^−1^ while the applied voltage was 10 kV in relation to the polymer concentration. The electrospinning process was carried out at ambient conditions. The nanofiber mat was obtained in a grounded plate collector with tip-to-collector distance (TCD) of 15 cm. After electrospinning, the electrospun PBz (ES-PBz) nanofiber mat was set aside at ambient conditions for 24 h to vaporize the remaining solvent prior to the post-electrospinning thermal treatment. Then, the nanofiber mat was thermally-crosslinked in an air-circulated drying oven (Deng Yng, DH 400) for sequential thermal treatment with temperatures of 80 °C, 120 °C, 160 °C, 200 °C, and lastly, 240 °C, each for 1 h. Then, the thermally cured mat was cooled down to room temperature inside the oven and then, removed from the collector plate.

### 2.3 Direct sulfonation of nanofibers

A two-stage experiment was carried out for the direct sulfonation process of thermally-crosslinked ES-PBz nanofibers. The first stage of the experiment tested only the one-variable-at-a-time involving the concentration of the sulfonating agent, *viz.*, concentrated and diluted 5 M and 6 M H_2_SO_4_, while the other parameters such as the reaction time and temperature were not changed. This part was conducted to establish the most appropriate concentrations of sulfuric acid that effectively modified the surface properties of the nanofibers. Direct sulfonation was conducted according to the procedure described in the work of Esmaielzadeh and Ahmadizadegan.^23^ The nanofiber mat samples were prepared (approx. 1 cm × 1 cm) from the thermally-crosslinked electrospun PBz nanofibers. Then, the samples were separately immersed in different concentrations of sulfuric acid (5 M, 6 M, and concentrated H_2_SO_4_) at room temperature for 72 h. The ES-PBz samples were removed from sulfuric acid and washed with deionized water repeatedly to remove the residual sulfuric acid until the pH of wash water was > 5. Subsequently, the samples were oven dried (Deng Yng, DH 400) at a temperature of 50 °C for 24 h.

In the first stage, concentrated sulfuric acid was effective in the direct sulfonation of the nanofiber mat samples based on the initial characterization methods. In the second stage of the sulfonation process, variable reaction time was investigated to determine the mechanism and kinetics of reaction. For this stage, the reaction time was varied from 3 h to 6 h and up to only 24 h using concentrated H_2_SO_4_. The same sulfonation procedure was used. Additional post-washing treatment was applied to the membrane samples after sulfonation, as reported from the works of Huang *et al.*^20^ In the acetone/water (1/1) (v/v) mixture, the sulfonated membrane samples were immersed for 5 min. Then, the samples were removed from the acetone/water mixture and immersed in pure acetone for 10 min. Subsequently, the samples were oven dried (Deng Yng, DH 400) at a temperature of 50 °C for 24 h.

### 2.4 Characterization of the electrospun nanofibers

The characterization of the electrospun nanofibers after sulfonation were conducted to evaluate the resulting properties by comparing the non-sulfonated electrospun nanofibers with the samples that undergo the sulfonation reaction. This is to determine if functionalities of the thermally-crosslinked ES-PBz nanofibers were modified or enhanced by the chemical treatment. The samples for testing were prepared according to the methods of characterization that were conducted. For FTIR, a very tiny sample was loaded in the transmission mode after pressing into a KBr disc while ATR was used in conjunction with FTIR to directly examine the portion of the nanofiber mat surface without further preparation. A 5 mm × 10 mm nanofiber mat sample was prepared for mounting and insertion into the instrument to conduct XPS analysis while almost similar size of the dried sample was placed on a carbon tape and gold was coated on the non-conductive nanofiber mat sample for SEM-EDX applications. Surface chemical characterization was performed with an X-ray photo electron spectrometer (XPS) (VG Microtech MT-500 ESCA) analysis to determine the change in the surface chemistry of the material. The resolution of the sub peaks was performed using the least-squares peak analysis software XPS PEAK 95 version 3.0. Attenuated total reflectance (ATR) sampling with Fourier-transform infrared (FTIR) spectra of the electrospun PBz nanofiber mat samples after the sulfonation treatment were obtained using a PerkinElmer Spectrometer at the wavenumbers between 400 cm^−1^ to 4000 cm^−1^. SEM-EDX analysis was carried out with a Scanning Electron Microscope (SEM) (FEI Helioz Nanolab 600i, Eindhoven, The Netherlands) with Energy Dispersive X-ray Spectroscopy (EDX) (Oxford Instrument X-Max, Abingdon, U.K.) at the Advanced Device and Materials Testing Laboratory (ADMATEL) of DOST. The water contact angle (WCA) of the electrospun membranes were measured using the water contact angle meter (First Ten Angstroms (FTA) Model: FTA 1000 B) with water drops of about 5 μL under the ambient conditions to evaluate the surface wettability of the electrospun nanofiber surfaces. The contact angle data were obtained from the average of three replicates from five measurements of the samples mounted on specimen glass. The pH level of the water used for determining the contact angle was adjusted from neutral to acidic and basic to further test the surface property.

The ion exchange capacity (IEC) was determined using a modified back titration procedure described in the work of Huang *et al.*^20^ Prior to the back titration procedure, the nanofiber mat samples were prepared by neutralization in 0.01 M sodium hydroxide aqueous solution in the sample to NaOH ratio of 0.025 g/10 mL for 72 h. This fully converted the sulfonated mat samples into their sodium salt form. Then, diluted sulfuric acid with the concentration of 0.003 M was employed to back titrate the NaOH aqueous solution that was partially neutralized by the sulfonated samples. The neutralization point in the back titration was predicted by using a universal indicator. The volume of sulfuric acid used in the titration was used for obtaining the IEC of the samples using eqn (1):^20^

1 IEC = *VN*/*m*_dry_

where IEC (meq g^−1^) is the ion exchange capacity (on a dry sample weight basis), *V* (mL) is the volume and *N* (mol L^−1^) is the normality of the sulfuric acid titrating solution, and *m*_dry_ (g) is the dry mass of the nanofiber mat. The pH of the NaOH solution used for soaking the samples was measured before and after soaking to determine the change in the basicity of the solution.

## 3. Results and discussion

### 3.1 Thermal crosslinking of the electrospun PBz nanofibers

Post-electrospinning treatment is an important thermal curing process for PBz as a physical method of modification prior to the next step functionalization. This curing process applied to the electrospun nanofibers uses sequential thermal treatment and causes improvement in the properties. During thermal curing, the oxazine ring opens by itself without hardeners or a strong acid catalyst.^6,8^ The modified nanofiber surface is produced due to the crosslinked fiber structures.^24^ Crosslinking is a chemical modification treatment of a nanofiber-based mat, which improves the mechanical properties, compactness, and chemical stability.^17^ In PBz, the cured material possesses low crosslinking density and exhibits near-zero shrinkage due to the removal of strain from ring opening polymerization.^2^

**Fig. 1:**
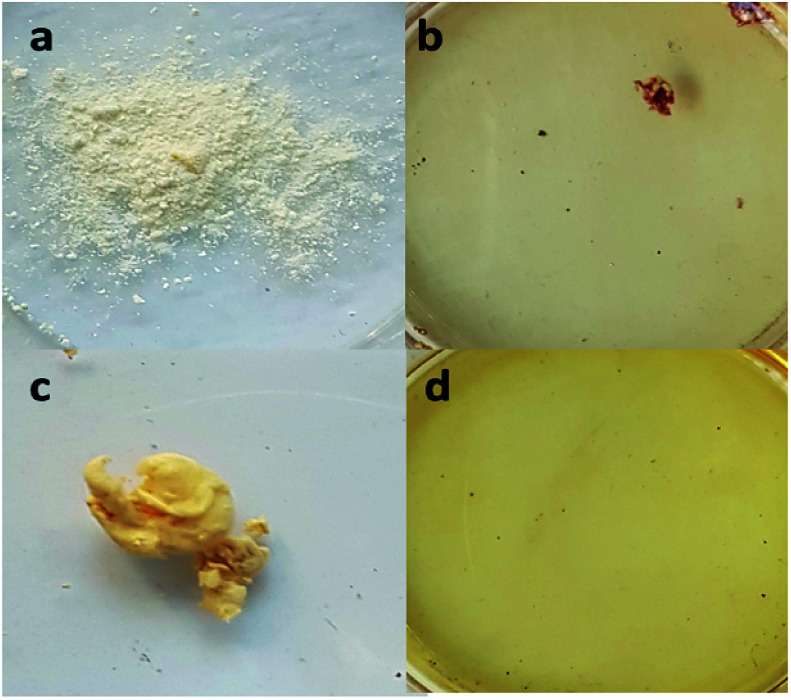
Solubility of (a) powdered PBz, (b) after soaking in H_2_SO_4_, (c) sample of the as-spun nanofibers of PBz, and (d) after soaking in H_2_SO_4_.

Direct sulfonation process uses sulfuric acid as one of the commonly used sulfonating solvents. The desired outcome for the sulfuric acid reaction during sulfonation is the introduction of sulfonic groups in the nanofibers to a certain degree.^25^ For the electrospun nanofibers that were thermally cured, the sulfonate group linkage could endow special features to produce functional nanofibers. To show the effect of using concentrated H_2_SO_4_ as the solvent in the sulfonation reaction, both powdered PBz and the as-spun nanofibers of PBz were tested by soaking in the acid. After less than 5 min in H_2_SO_4_, the powdered PBz completely dissolved, as shown in Fig. 1a and b. For the as-spun nanofibers, which was set aside for 24 h to remove the residual solvent and then soaked in H_2_SO_4_, also resulted in complete dissolution, as shown in Fig. 1c and d.

These results showed the strength of H_2_SO_4_ as a sulfonating agent that affects the stability of the material. Through these solubility tests, the concentration of sulfuric acid was identified as a critical factor in the sulfonation reaction for electrospun nanofibers. To investigate the sulfonation reaction with the thermally-crosslinked PBz nanofiber, the first variable of concern is the acid concentration.

### 3.2 Effect of H_2_SO_4_ concentration on the nanofiber mat

**Fig. 2:**
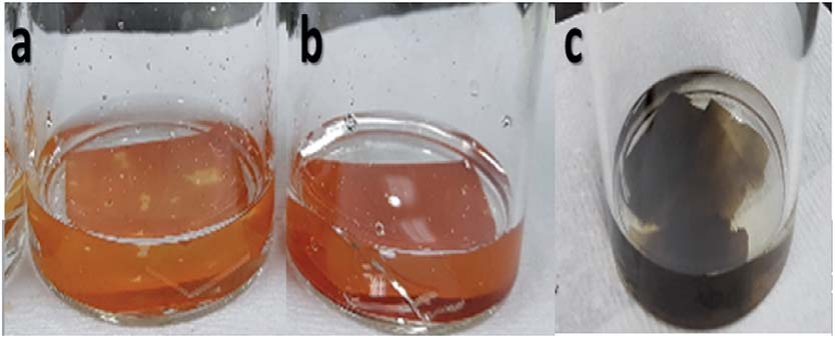
Change in color of the nanofiber samples during sulfonation with different concentration of sulfuric acid (a) 5 M, (b) 6 M, and (c) concentrated sulfuric acid.

The sulfonation of the electrospun nanofibers was undertaken under ordinary conditions with sulfuric acid as the sulfonating agent. Based on a previous study, using sulfuric acid at room temperature, the reaction occurred at a slower pace, so a long reaction time was needed.^20^ The reaction time was initially set to 72 h with the concentration of sulfuric acid as the only variable from the lower concentration of 5 M and 6 M to concentrated H_2_SO_4_ (97%) during the initial experiment of the sulfonation process. As shown in Fig. 2, the change in the color of the nanofiber mat samples from light brown to reddish brown and eventually to black were observed with the increase in the concentration of sulfuric acid but there was no effect on the stability of the nanofibers. The color change was a clear indication that a reaction between the electrospun nanofiber and the sulfonating agent took place but the dissolution of nanofibers was not seen.

**Fig. 3:**
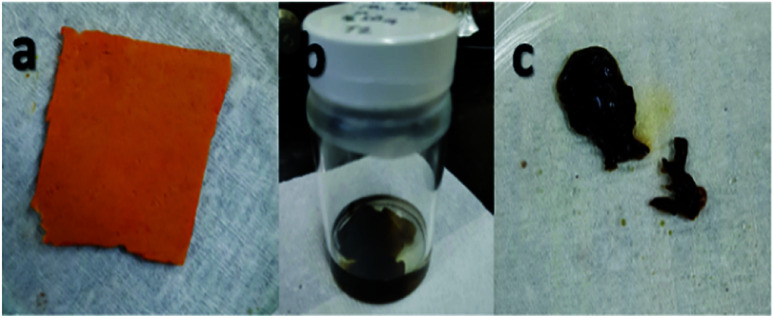
Electrospun nanofiber samples (a) before sulfonation, (b) during sulfonation with concentrated sulfuric acid at 72 h, and (c) after washing, which formed into a gel.

During the first stage it was found that using concentrated sulfuric acid, the sample formed into a gel during washing with deionized water, as shown in Fig. 3. This result indicated that using concentrated H_2_SO_4_ for a longer reaction time of 72 h also resulted in longer exposure to the reaction and affected the dimensional stability of the sample even if sulfonation was achieved.

From these observations, the reaction time was shortened to only 24 h for the second stage with concentrated sulfuric acid as the only concentration used to avoid the occurrence of the same condition. The second stage of the experiment involving the sulfonation process established the interactions of the acid group with the nanofibers, which also exhibited the beneficial effect of crosslinking.

### 3.3 Mechanism and kinetics of functionalization by sulfonation

**Fig. 4:**
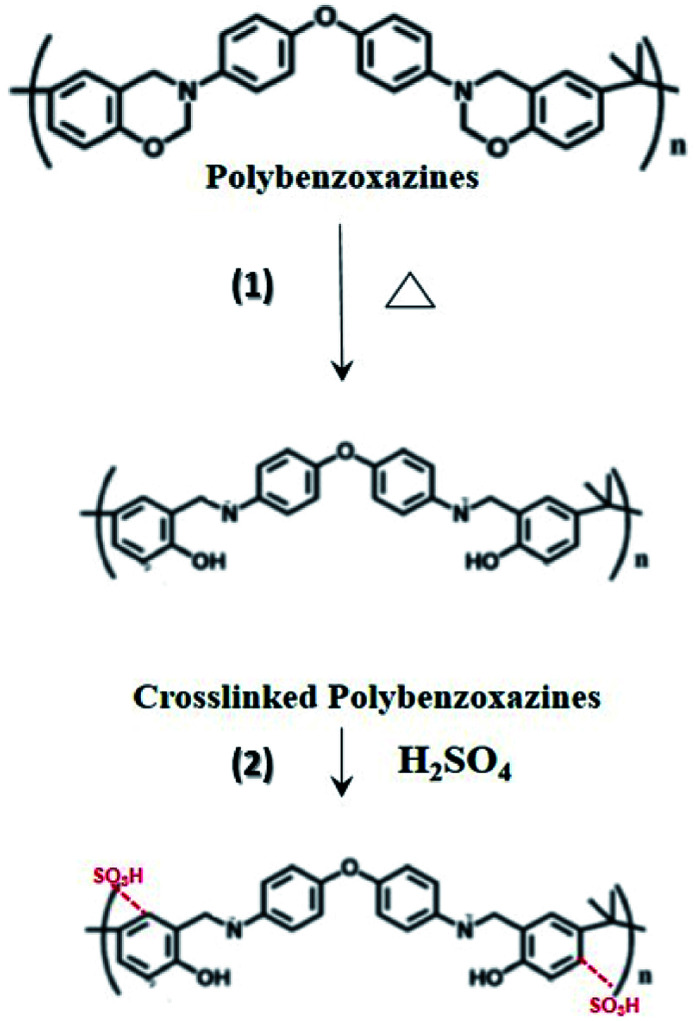
Proposed scheme of functionalization by step (1) thermal treatment, followed by step (2) sulfonation reaction of the electrospun PBz nanofibers.

In sulfonation, the reaction of the sulfonating agent with the electrospun nanofibers occurred where sulfonic acid was added to the aromatic ring by electrophilic substitutions.^20,26^ Based on the previous works, the reaction was found to be second order with respect to SO_3_ and first order with respect to aromatic compounds.^27^ The substitution preferentially occurred at the ortho-position of the aromatic ring in the repeating unit of PBz, as shown in Fig. 4. Using sulfuric acid as the sulfonating agent under room temperature, only one sulfonic acid (–SO_3_H) group attached to each of the repeating units since only first type substitution occurs because of the low energy barrier of this sulfonation condition.^20^

On using sulfuric acid as the sulfonating agent, the active site is due to the electron density of the site. The sulfonation reaction at low temperature only occurs in one of the aromatic rings since the electron density of the other aromatic ring in the repeating unit is relatively low due to the neighboring group.^20^ In addition, the crosslinking reaction in the polymerization of benzoxazines prior to sulfonation proceeds through the electrophilic attack of the ring-opened benzoxazine structure, resulting in the available *ortho* (more activated, preferred site) and *para* positions of the neighboring phenolic ring for electrophilic substitution during the sulfonation reaction.^28^ Although there were no previous works that confirmed the sulfonation of nanofibers PBz, this study proposed that the mechanism of sulfonation reaction is confined to the first type substitution, as shown in Fig. 4.

**Fig. 5:**
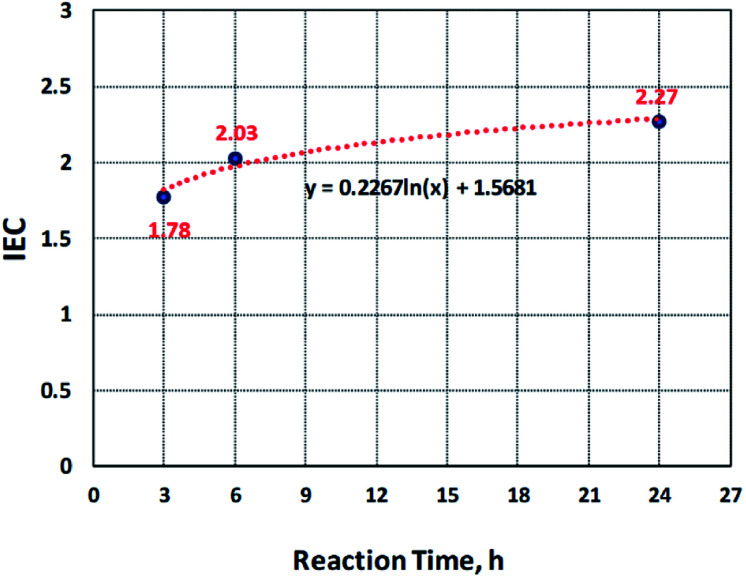
Time dependence of sulfonation reaction of PBz nanofibers.

IEC is the number of moles of the sulfonate groups (ions) present per gram of the polymer.^29^ The ion exchange capacity of the sulfonated electrospun nanofibers of PBz was determined by acid–base titration and compared with the samples sulfonated at different reaction times (3 h, 6 h, and 24 h). At 3 h of the reaction time, the IEC measured was to be 1.78 while at 6 h, it increased 2.03 and obtained the IEC value of 2.27 after 24 h, as presented in Fig. 5. These results showed that increasing the reaction time also increased the IEC values where the IEC value for the sample sulfonated at the reaction time of 24 h has the highest value. This confirmed that the sulfonate groups were incorporated in the electrospun nanofibers during the direct sulfonation reaction and the rate of reaction increases with increasing reaction time. The pH of NaOH before and after soaking the samples also confirmed this with the change in the pH values from 9.7 to 8.8.

The kinetics of sulfonation reaction of electrospun PBz nanofibers was evaluated by determining the correlation of reaction time with the amount of sulfonate groups present in the nanofibers. It exhibited a non-linear correlation and observed that the data is logarithmically related, as shown in Fig. 5. The level of IEC increased steadily up to 18 h and reached a plateau between 18 to 24 h of sulfonation. From the trendline, the derived empirical equation of IEC as a function of reaction time was obtained as:

2 *y*(IEC) = 0.2267 ln *x*(*t*) + 1.5681

where *y* is the IEC value and *x* is the reaction time with a correlation coefficient of 0.9797, indicating strong relationships between the reaction time and the IEC value.

However, based on these results, the kinetics of reaction at ordinary conditions confirmed that only the slow reaction took place. The kinetics of reaction with IEC as a function of reaction time exhibited a logarithmic trendline where the rate of change in IEC with the reaction time increased rapidly and then, almost level out or show no significant change in the reaction time between 18 h to 24 h. Further investigation could be undertaken with reaction temperature as another variable.

### 3.4 Surface chemistry

**Fig. 6:**
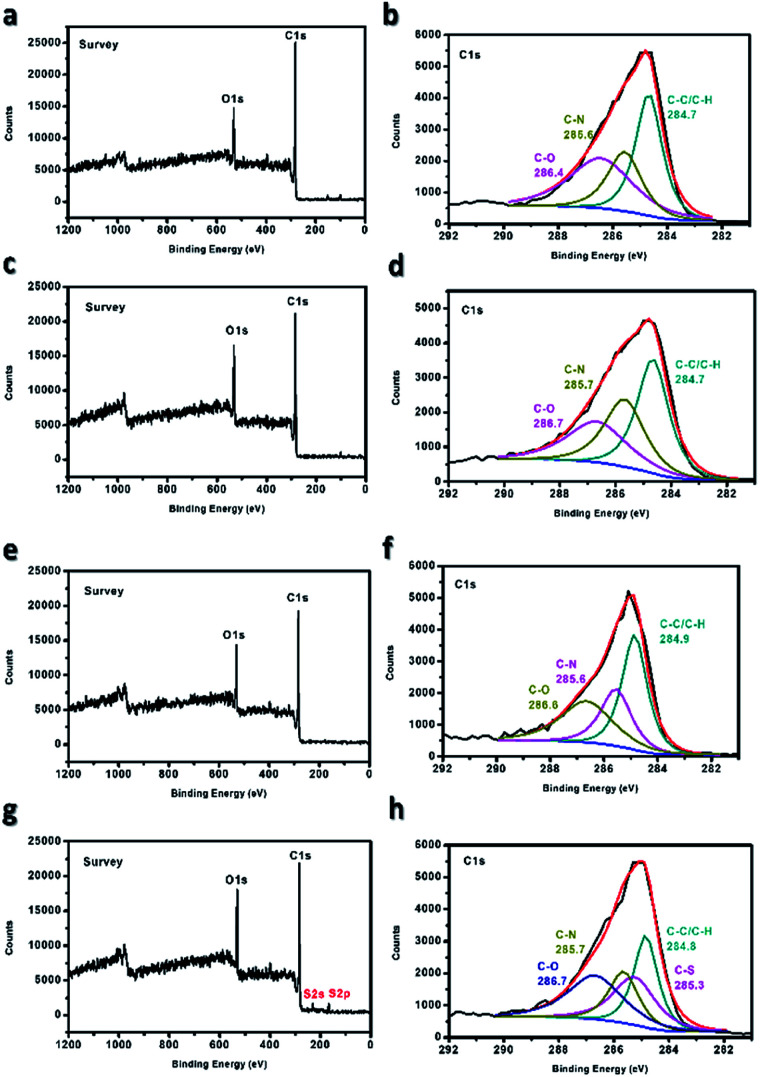
XPS spectra of ES-PBz (a and b) non-sulfonated, (c and d) sulfonated using 5 M, (e and f) sulfonated using 6 M, and (g and h) sulfonated using concentrated H_2_SO_4_.

The sulfonation of the electrospun PBz nanofibers was confirmed using XPS to determine the change in the surface chemistry that occurred in the material. From Fig. 6(a and b), which exhibited only the O 1s and C 1s regions on the survey, the non-sulfonated ES-PBz exhibited three characteristic peaks in the C 1s region at the binding energies of 284.7 eV, 285.6 eV, and 286.4 eV, which were attributed to C–C/C–H, C–N, and C–O, respectively. From the XPS spectra of the other ES-PBz samples, as shown in Fig. 6(c–h), which undergo sulfonation at different concentrations of sulfuric acid (5 M, 6 M, and concentrated), only the nanofibers sulfonated using concentrated sulfuric acid showed a new peak in the S 2p region. This sample exhibited a new characteristic peak at the binding energy of 285.3 eV associated with C–S owing to the sulfonate group introduced on the surface of the ES-PBz. While, for the sulfonation of samples using lower concentrations of 5 M and 6 M H_2_SO_4_, the same characteristic peaks were observed similar to the non-sulfonated ES-PBz without the presence of the characteristic peak attributed to C–S. Based on the XPS analysis, only the nanofiber samples that were sulfonated using concentrated sulfuric acid, were we to incorporate the sulfonate groups onto the surface of the nanofibers.

Further confirmation of sulfonation was obtained from the elemental composition of ES-PBz samples and their relative atomic percentages, as listed in Table 1. Sulfur (S 2p) from the sulfonating agent was only present in the sulfonated ES-PBz with the relative atomic percentage of 1.9%, corresponding to the sulfonic acid group incorporated in the sample during the sulfonation reaction. This confirmed the functionalization of ES-PBz by the sulfonation reaction using sulfuric acid.

**Table 1:** Elemental composition of non-sulfonated and sulfonated ES-PBz and the relative atomic percentages based on the XPS spectra

at%	Non-sulfonated	Sulfonation H_2_SO_4_ concentration		
		5 M	6 M	Concentrated (97%)
O 1s	16.9%	19.8%	17.9%	15.3%
C 1s	83.1%	80.2%	82.1%	82.8%
S 2p	—	—	—	1.9%

### 3.5 Structural composition

**Fig. 7:**
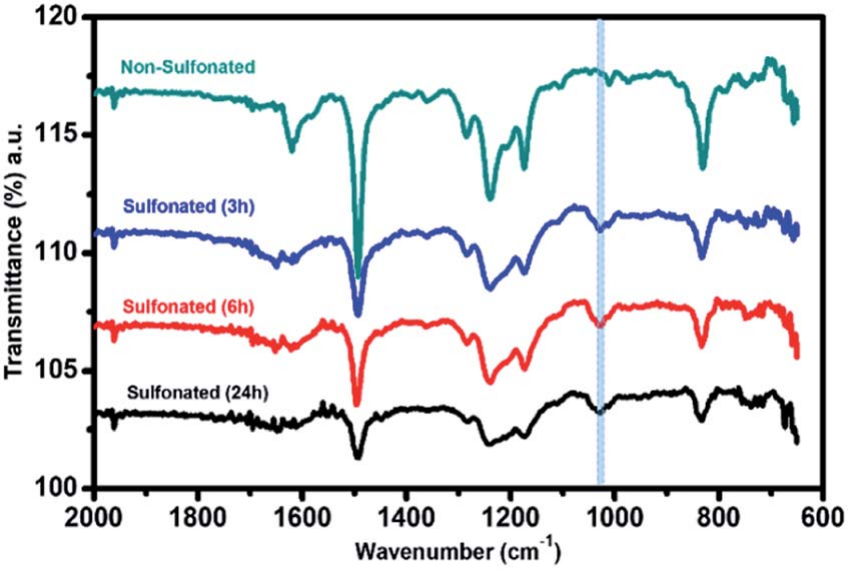
FTIR spectra of the electrospun PBz nanofibers and sulfonated electrospun PBz nanofibers at diluted concentration of 5 M and 6 M, and concentrated sulfuric acid after 72 h.

In order to further confirm the sulfonation reaction with the crosslinked PBz nanofibers, FTIR spectroscopy was carried out to validate the structural composition of the material in comparison with the results of electron spectroscopy. As shown in Fig. 7, the characteristic peaks associated with the benzoxazine structure at 1230–1235 cm^−1^ (asymmetric stretching of C–O–C), 1330–1340 cm^−1^ (CH_2_ wagging in the closed benzoxazine ring), and 1495–1510 cm^−1^ (tri substituted benzene ring) were present.^9^ For the ES-PBz that undergoes sulfonation as compared to the non-sulfonated ES-PBz, a new peak at 1025–1035 cm^−1^ was observed, which corresponds to the symmetric stretch of the sulfonate group (SO_3_). The presence of sulfonate (SO_3_) group is the result of functionalization of the nanofibers. From the spectra, it was observed that the intensity of the new peak assigned to the sulfonate group was more prominent in the sample that was sulfonated using concentrated sulfuric acid for 24 h as compared to the sulfonated samples at diluted concentration of 5 M and 6 M sulfuric acid at 72 h. This could be the reason for the absence of the sulfonate group, as indicated by the C–S in the XPS analysis of the sulfonated samples at lower concentrations.

**Fig. 8:**
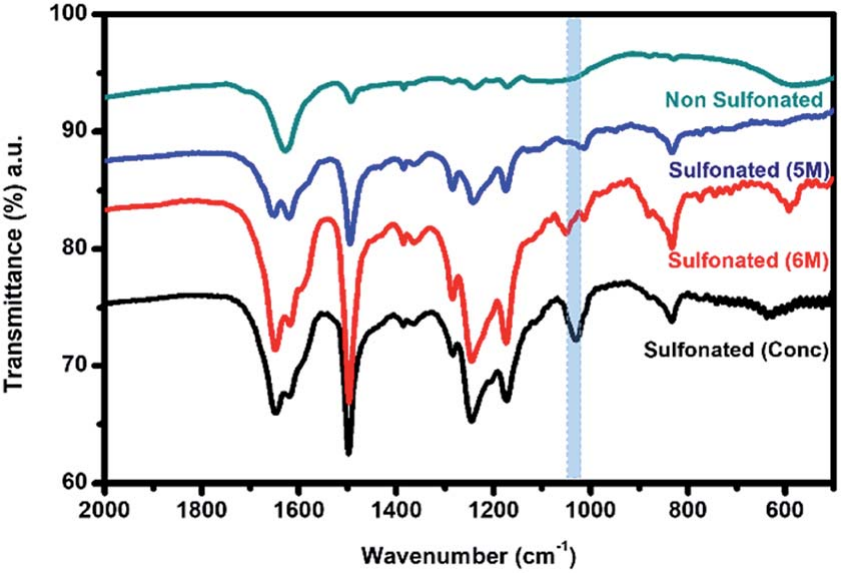
ATR-FTIR spectra of the electrospun PBz nanofiber mats sulfonated with concentrated sulfuric acid at varying reaction times of 3 h, 6 h, and 24 h.

For the second stage of sulfonation using only concentrated sulfuric acid with varied reaction times of 3 h, 6 h, and 24 h, attenuated total reflectance (ATR) with Fourier transform infrared (FTIR) spectroscopy was used to directly measure the change in the surface chemistry on the nanofiber mat surface and to validate the sulfonation process of the samples at lower reaction times. The ATR-FTIR spectra in Fig. 8 showed that all the three samples at different sulfonation reaction times of 3 h, 6 h, and 24 h exhibited a new peak in the range of 1025–1035 cm^−1^ with almost similar intensity. This characteristic absorption band corresponds to the symmetric stretch of the sulfonate group (SO_3_), indicating that the sulfonation reaction took place on the samples' surface. This also validated the results of XPS analysis that sulfonate group was only present in the sulfonation reaction using concentrated H_2_SO_4_ with the strong intensity of the peak assigned to SO_3_. Thus, proving that the sulfonation reaction under ordinary conditions with shorter reaction time (3–24 h) resulted in surface modification of the electrospun PBz nanofibers.

### 3.6 Surface composition, microstructure, and morphology

**Fig. 9:**
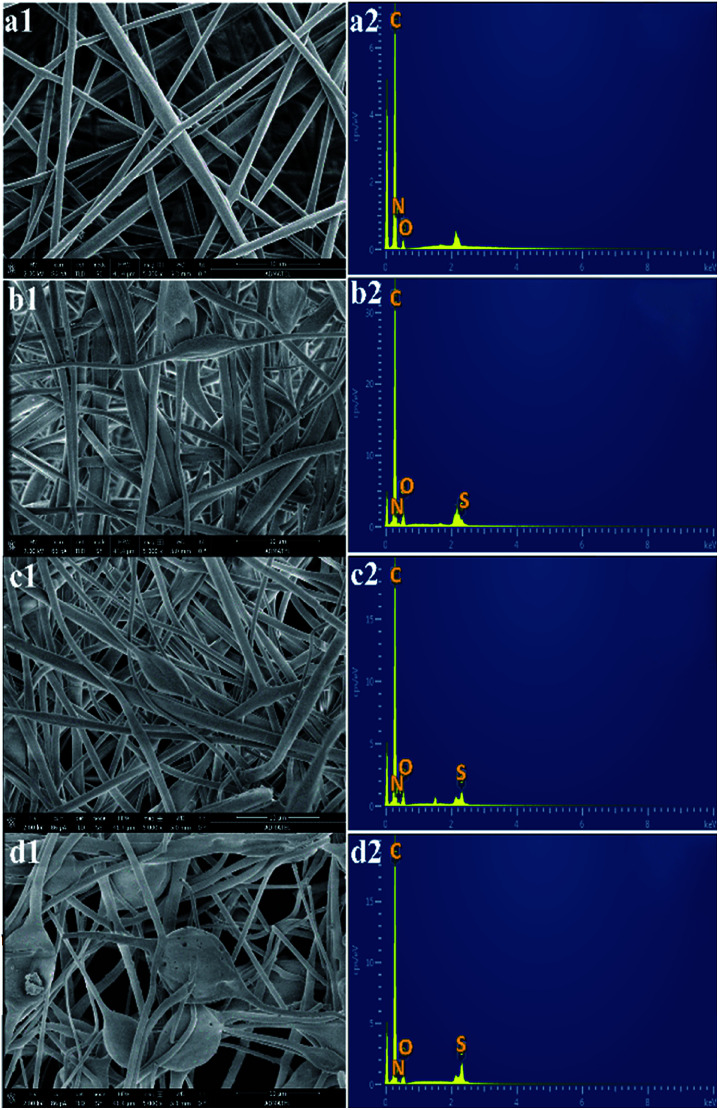
SEM micrographs and EDX spectra of the electrospun nanofibers: (a1 and a2) non-sulfonated ES-PBz; (b1 and b2) sulfonated ES-PBz at 3 h; (c1 and c2) sulfonated ES-PBz at 6 h; and (d1 and d2) sulfonated ES-PBz at 24 h: (magnification: 5000×; scale bar: 10 μm); other peaks without labels in the spectra are due to Au, which was used as the coating material.

The SEM images of the non-sulfonated electrospun PBz nanofibers in Fig. 9a1 show a uniform fiber morphology with an average fiber diameter of 2.668 ± 0.987 μm and an evenly distributed fiber diameter between 1.3–3.3 μm. After undergoing the sulfonation process for 3 h, 6 h, and 24 h, the structure of the randomly-oriented continuous, interconnected fiber networks were still evident and intact, as shown in Fig. 9b1–d1. The sulfonation reaction involving concentrated sulfuric acid did not show any drastic effect on the nanofiber's structural stability except for very few broken fibers in the electrospun nanofibers treated for 24 h reaction time. It has been studied in a previous work that the membrane samples became more brittle when sulfonated at a higher concentration of the acid (>0.2 mol L^−1^).^25^ This could be the reason for the few broken fibers in the mat samples. In addition, a rougher surface was observed in the nanofibers, which could be the result of the wetted fibers with the sulfonate group that is attached to the nanofibers' surface after the sulfonation reaction.

The EDX spectra were then analyzed to determine the overall chemical composition and the distribution of the chemical elements of interest in the sulfonated electrospun nanofibers. The EDX spectra of the non-sulfonated ES-PBz, as expected, show no presence of the sulfur element in the nanofibers whereas the carbon element predominantly appeared, as shown in Fig. 9a2. The EDX spectra have peaks of carbon, nitrogen, and oxygen only. Based on the EDX spectra, all the sulfonated electrospun nanofibers exhibited sulfur peaks along with the peaks assigned to carbon, nitrogen, and oxygen (Fig. 9b2–d2). In addition, the relative peak heights of the chemical elements present in the polymer were clearly correlated with the respective amount present in the nanofibers. The sulfur peak present in the EDX spectra of the samples sulfonated at shorter reaction times (3 h and 6 h) is a relatively short sulfur peak in the nanofibers as compared to the sulfur peak of the sulfonated sample with longer reaction time of 24 h. It could be deduced from these results that qualitatively, the concentration of the sulfonate groups present in the nanofibers is directly proportional to the reaction time, which resulted in a higher degree of sulfonation using concentrated sulfuric acid.

### 3.7 Water contact angle

Contact angle is an important functional property of a material. The change in the contact angle indicates a modification in the surface chemistry.^20^ If the sulfonation reaction was successful in incorporating sulfonate groups on the nanofibers' surfaces, then there is an expected change in the hydrophobicity of the ES-PBz nanofibers. Non-sulfonated ES-PBz nanofibers are hydrophobic with a water contact angle of 130° based on the previous work of Parreño *et al.*^16^ The crosslinking that occurred in the PBz nanofibers during thermal treatment is known to be an effective method to change the morphology as well as the surface properties of the polymers.^20^ However, pure PBz nanofibers retained their hydrophobicity even after undergoing post-electrospinning thermal treatment.

**Fig. 10:**
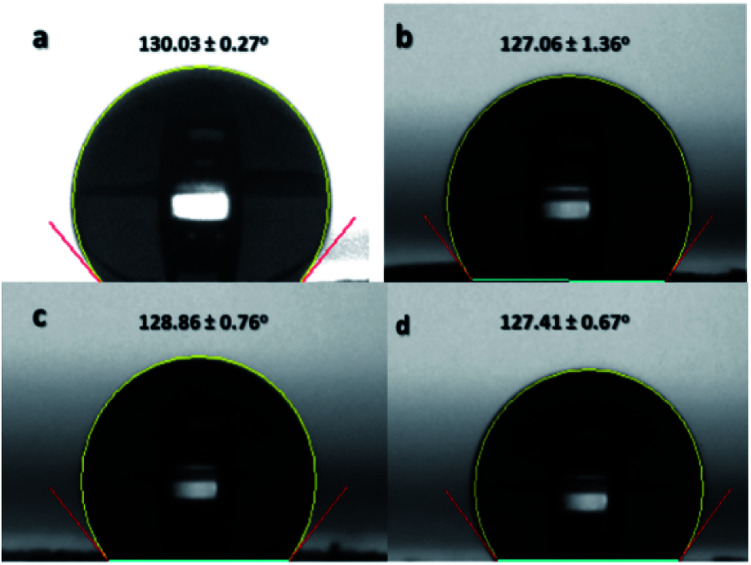
Water contact angle of (a) non-sulfonated ES-PBz, (b) sulfonated ES-PBz after 3 h, (c) sulfonated ES-PBz after 6 h, and (d) sulfonated ES-PBz after 24 h of reaction. The values are means ± standard deviations with three replicates taken per data point.

In this study, the water contact angles of the nanofiber mat samples were determined to know if there was an effect on the surface chemistry, as indicated in the IR and electron spectroscopy after the sulfonation process. The contact angles were measured for the non-sulfonated and sulfonated samples at different reaction times for comparison. The results showed that the non-sulfonated, thermally-crosslinked ES-PBz retained its hydrophobicity with a contact angle of 130.03°, as shown in Fig. 10a. For the sulfonated samples, the contact angles of the samples were 127.06°, 128.86°, and 127.41° after 3 h, 6 h, and 24 h of reaction, respectively, as indicated in Fig. 10b–d. The incorporation of sulfonate groups onto the surface of the nanofibers did not alter the surface chemistry of the nanofibers. The reason for this is the presence of inter- and intra-molecular hydrogen bonding within the PBz structures, which prevented the hydroxyl groups from interacting with the water molecules.^2^ The ultralow surface-energy of the crosslinked PBz nanofibers is the result of the hydrogen bonding, which is mainly responsible for the surface properties.^22^

**Fig. 11:**
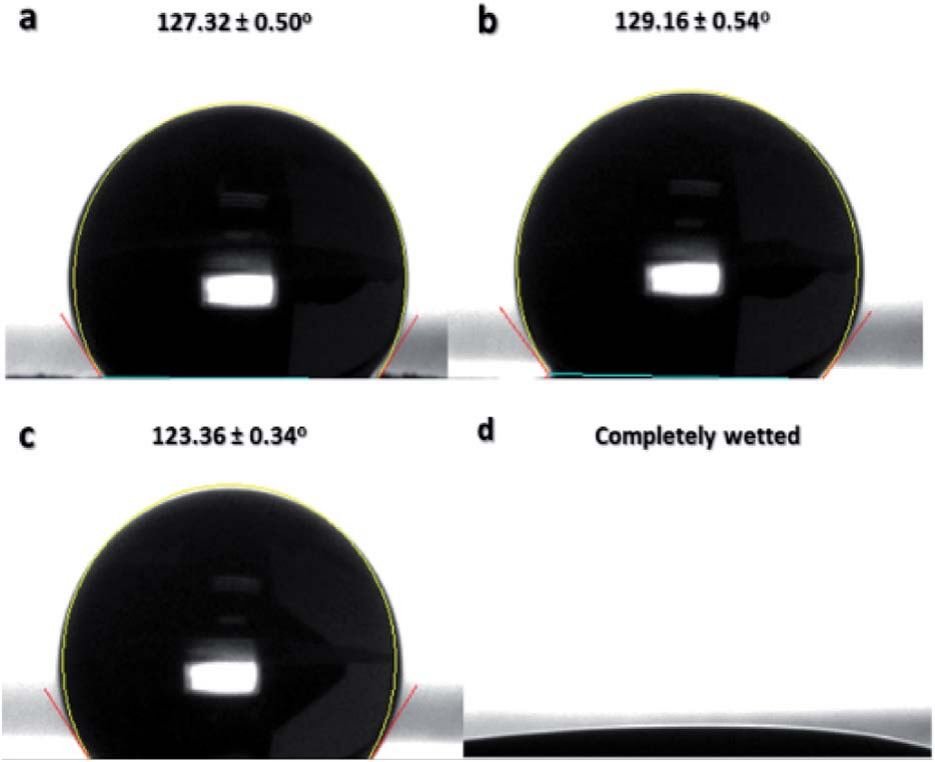
Water contact angle of ES-PBz nanofibers sulfonated for 24 h when pH level is (a) 3, (b) 6, (c) 12, and (d) 14. The values are means ± standard deviations with three replicates taken per data point.

Surface wettability of the sulfonated samples at 24 h reaction time was further characterized by adjusting the pH level of water from acidic to basic to confirm another surface property of the thermally-crosslinked ES-PBz. From a previous study, it was found out that crosslinked PBz has an intrinsically stimuli responsive characteristic due to the hydrogen bonding, which can be broken by changes in pH level of water.^22^ The pH level of water used for the measurements of water contact angle were adjusted to acidic (pH: 3 and 6) and basic (pH: 12 and 14) to examine this surface property. At acidic pH of water, the contact angles at pH 3 and 6 were 127.32° and 129.16°, respectively, as shown in Fig. 11a and b. At basic pH of 12 in Fig. 11c, the contact angle measured was 123.36°, which is still hydrophobic and similar to the contact angle of acidic water. However, when the pH level was further adjusted to 14, the sample became completely wetted, where the wettability changed from hydrophobic to hydrophilic, as shown in Fig. 11d.

This is the validation of the surface properties of the ES-PBz nanofibers, which switch to hydrophilic when the pH of water changes to highly basic. This functional property of the sulfonated PBz nanofibers has potential applications in oil–water separation as they have stimuli responsive surfaces and combined enhancement obtained from the crosslinking and electrospun fiber structure and morphology.

### 3.8 Thermal stability

**Fig. 12:**
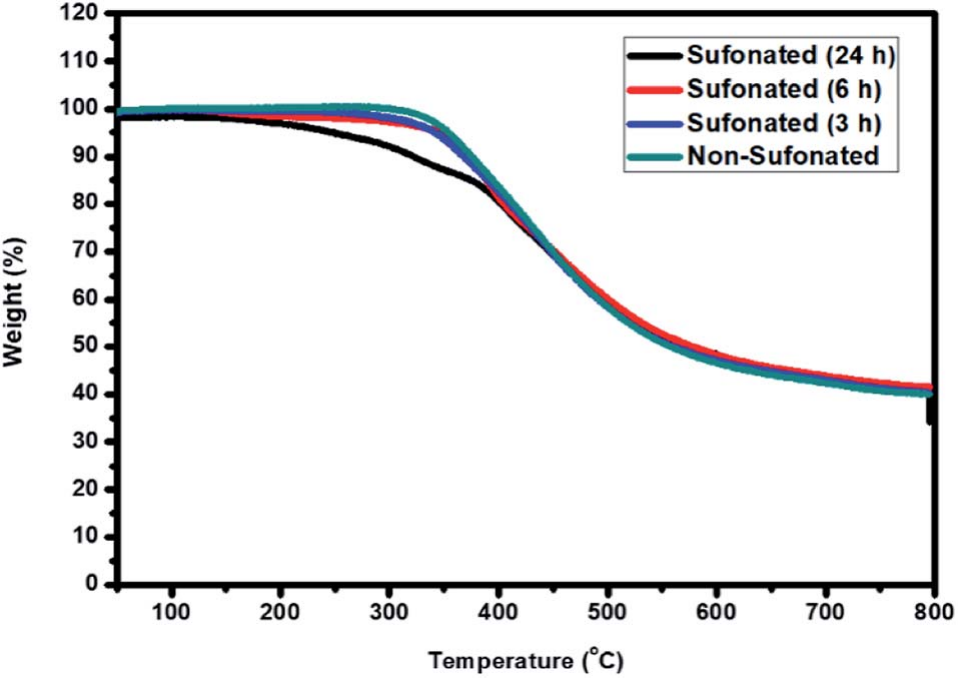
Thermal stability of the PBz nanofibers sulfonated at 3 h, 6 h, and 24 h reaction time as compared to the non-sulfonated PBz nanofibers.

The thermal stability of the sulfonated electrospun nanofibers of PBz at different reaction times of 3 h, 6 h, and 24 h were compared to the non-sulfonated electrospun nanofibers to determine the influence of the sulfonation reaction on the nanofibers' stability. Based on the TGA thermograms, as shown in Fig. 12, the non-sulfonated and sulfonated samples showed almost the same thermal resistance with the onset temperature of degradation at 275 °C except for the sulfonated sample at the reaction time of 24 h where it started its degradation at a relatively lower temperature of 220 °C. The lower temperature of degradation of the sample nanofibers sulfonated at 24 h reaction time could be attributed to the higher amount of sulfonic acid group incorporated in the nanofibers for a longer sulfonation reaction compared to 3 h and 6 h of reaction time. Between 220–375 °C, there is a steep, massive weight loss due to the decomposition of sulfonic acid. By increasing the temperature, the weight loss reaches a plateau, where no further degradation occurs at 500 °C. This thermal behavior confirmed that a higher sulfonation degree was achieved with longer reaction time but it affected the onset temperature of degradation. Based on these results, sulfonation at the reaction time of 6 h is the most appropriate for the retention of thermal stability.

## 4. Conclusions

This study provides a new perspective on the functionalization of nanofibers of PBz prepared by the electrospinning process by direct sulfonation reaction using sulfuric acid as the sulfonating agent. The crosslinking of PBz nanofibers by post-electrospinning thermal treatment showed its contributing factor in accommodating the succeeding sulfonation reaction. The mechanism of incorporating the functional groups onto the surface of the PBz nanofibers was analyzed and proposed as first type substitution of sulfonic acid in the aromatic ring, which was effectively carried out using concentrated sulfuric acid. The kinetics of the sulfonation process exhibited a logarithmic correlation where the rate of reaction is time dependent. But with low energy barrier under ordinary sulfonation conditions, it occurred at a slower pace and reached a plateau between the reaction time of 18 to 24 h. The microstructures and morphology after undergoing the sulfonation process were unchanged but had few broken fibers and rougher nanofibers due to the wetted fiber surfaces with the added sulfonate groups. The surface wettability of the nanofibers after sulfonation retained the hydrophobicity and revealed the intrinsic stimuli responsive property of the nanofibers on changing the pH of water to basic. Thermal stability was retained for 3 h and 6 h reaction time in comparison to the non-sulfonated nanofibers. Thus, a simple and easy yet effective functionalization method that can be carried out on crosslinked PBz nanofibers without compromising their form and stability has potential for the targeted applications although further study on other parameters of the sulfonation reaction is needed to achieve the desired results.

## Conflicts of interest

There are no conflicts to declare.

## Supplementary Material
